# Clustering of antipsychotic-naïve patients with schizophrenia based on functional connectivity from resting-state electroencephalography

**DOI:** 10.1007/s00406-023-01550-9

**Published:** 2023-02-02

**Authors:** Karen S. Ambrosen, Fanny Fredriksson, Simon Anhøj, Nikolaj Bak, Edwin van Dellen, Livia Dominicus, Cecilie K. Lemvigh, Mikkel E. Sørensen, Mette Ø. Nielsen, Kirsten B. Bojesen, Birgitte Fagerlund, Birte Y. Glenthøj, Bob Oranje, Lars K. Hansen, Bjørn H. Ebdrup

**Affiliations:** 1 Center for Neuropsychiatric Schizophrenia Research (CNSR) and Center for Clinical Intervention and Neuropsychiatric Schizophrenia Research (CINS), Copenhagen University Hospital, Mental Health Services CPH, Nordstjernevej 41, 2600 Glostrup, Denmark; 2 H. Lundbeck A/S, Valby, Denmark; 3 Department of Psychiatry, University Medical Center Utrecht, Brain Center Rudolf Magnus, Utrecht, The Netherlands; 4 Department of Clinical Medicine, Faculty of Health and Medical Sciences, University of Copenhagen, Copenhagen, Denmark; 5 Department of Psychology, University of Copenhagen, Copenhagen, Denmark; 6 Department of Applied Mathematics and Computer Science, DTU Compute, Technical University of Denmark, Kgs. Lyngby, Denmark

**Keywords:** Resting-state electroencephalography, Antipsychotic-naïve first-episode schizophrenia, Clustering, Functional connectivity, Psychopathology, Cognition

## Abstract

Schizophrenia is associated with aberrations in the Default Mode Network (DMN), but the clinical implications remain unclear. We applied data-driven, unsupervised machine learning based on resting-state electroencephalography (rsEEG) functional connectivity within the DMN to cluster antipsychotic-naïve patients with first-episode schizophrenia. The identified clusters were investigated with respect to psychopathological profile and cognitive deficits. Thirty-seven antipsychotic-naïve, first-episode patients with schizophrenia (mean age 24.4 (5.4); 59.5% males) and 97 matched healthy controls (mean age 24.0 (5.1); 52.6% males) underwent assessments of rsEEG, psychopathology, and cognition. Source-localized, frequency-dependent functional connectivity was estimated using Phase Lag Index (PLI). The DMN-PLI was factorized for each frequency band using principal component analysis. Clusters of patients were identified using a Gaussian mixture model and neurocognitive and psychopathological profiles of identified clusters were explored. We identified two clusters of patients based on the theta band (4–8 Hz), and two clusters based on the beta band (12–30 Hz). Baseline psychopathology could predict theta clusters with an accuracy of 69.4% (*p* = 0.003), primarily driven by negative symptoms. Five a priori selected cognitive functions conjointly predicted the beta clusters with an accuracy of 63.6% (*p* = 0.034). The two beta clusters displayed higher and lower DMN connectivity, respectively, compared to healthy controls. In conclusion, the functional connectivity within the DMN provides a novel, data-driven means to stratify patients into clinically relevant clusters. The results support the notion of biological subgroups in schizophrenia and endorse the application of data-driven methods to recognize pathophysiological patterns at earliest stage of this syndrome.

**Supplementary Information:**

The online version contains supplementary material available at 10.1007/s00406-023-01550-9.

## Introduction

The discovery of intrinsic large-scale brain networks that subserve complex human behaviors has led to several network models for schizophrenia. Echoing the deficits seen in the clinical phenotype of this severe neuropsychiatric syndrome, these models include a range of core neurocognitive networks [1–8]. Even though these models focus on different aspects of cognition and sensory processing, they all emphasize the role of the default mode network (DMN) in the development of severe psychopathology and cognitive deficits. We have previously reported alterations in the DMN using resting-state functional magnetic resonance imaging (fMRI) in antipsychotic-naïve first-episode patients [9] as well as neurochemical and functional abnormalities in areas involved in the DMN [10, 11] using samples partly overlapping with the samples included in the current study.

Originally, the DMN was described as a widespread set of regions throughout the association cortex that was de-activated during active tasks [3]. Building on this discovery of a unified set of regions with de-activation as a unifying feature, large-scale network models of psychosis largely focus on the connectivity between different large-scale networks such as DMN, the central-executive network and the salience network, and the association with psychiatric symptoms [5]. The relevance of this approach has been demonstrated in numerous studies including an earlier study from our group that showed abnormal functional connectivity between the DMN and auditory network [9]. In recent years, a growing body of evidence has supported the notion that the DMN is not a single, unified network but rather comprises multiple sub-networks associated with different cognitive functions [12]. This in turn has fostered a demand to study connectivity between key areas of the DMN in healthy participants with normal cognitive functioning and in mental illness in addition to studying the average signal across all regions of the DMN.

In the attempt to understand schizophrenia from a network perspective, studies on the association between DMN connectivity and clinical manifestations are of particular interest, and a growing body of evidence points toward abnormal DMN connectivity in schizophrenia patients [13]. Nevertheless, the results are conflicting in relation to directionality and magnitude, and the clinical implications of the aberrations in DMN connectivity are unclear. Variability across patient samples in terms of disease progression, medication status, and methodology may explain some of these differences. Currently, there is no consensus on how to best analyze EEG data with respect to functional connectivity analyses which impedes the comparison between studies [14]. To what extend the variability within DMN connectivity measured with EEG in antipsychotic-naïve first-episode patients may represent subgroups of schizophrenia patients is largely unexplored.

Most previous studies of intrinsic connectivity in clinical samples have been using resting-state fMRI, which provides an indirect measure of neuronal activity with high spatial resolution. Regardless of the methodological differences, resting-state electroencephalography (rsEEG) studies have revealed intrinsic large-scale networks, which are similar to the well-established networks in the fMRI literature [15]. Compared to fMRI, EEG is a direct measure of neural activity providing a superior temporal resolution, which may be of importance for the detection of subtle temporal abnormalities in network interactions hypothesized to underpin clinical symptoms. Furthermore, EEG measures are more easily implemented in the everyday clinic and lower in cost, and therefore, we opted for this modality in the current paper.

In this study, we first investigated group differences in the EEG functional connectivity within the DMN between antipsychotic-naïve, first-episode patients with schizophrenia and healthy controls (HC). Second, we applied a bottom–up approach using unsupervised machine learning (ML) to cluster patients based on functional connectivity within the DMN. We analyzed each frequency band separately to better handle our sample size, as well as to enhance interpretability of the results. Finally, as suggested by Marquand et al. [59], we confirmed the relevance of the identified subgroups by applying Support Vector Machines (SVM) to test if the subgroups were associated with cognitive and psychopathological measures. We hypothesize that we can discriminate subgroups based on the functional connectivity within the DMN measured with EEG, and that these subgroups have clinical relevance.

## Methods

### Participants

We included 37 antipsychotic-naïve, first-episode patients with schizophrenia and 97 matched HC recruited in two consecutive multimodal cohorts: The Pan European Collaboration on Antipsychotic Naïve Schizophrenia (PECANS, ClinicalTrials.gov Identifier: NCT01154829) and the Pan European Collaboration on Antipsychotic Naïve Schizophrenia II (PECANSII, ClinicalTrials.gov Identifier: NCT02339844) as previously described in [10, 11]. For full description, see www.ClinicalTrials.gov. The studies were approved by the Regional Danish Committee on Health Research Ethics (H-D-2008-088, H-3-2013-149). The patients were referred from in- and outpatient clinics in the Capital Region of Denmark. HC were recruited from the community of the Capital Region through online advertisement and were matched with respect to sex, age, and parental socioeconomic status. Inclusion criteria for patients were a diagnosis of psychosis spectrum according to ICD-10 or DSM-IVR and lifetime naïve to antipsychotic exposure. However, in the current study, we only included the patients with a F20.X diagnosis. Exclusion criteria for the patients were current drug-dependence (except for nicotine), organic brain damage, previous impact-related unconsciousness, contraindications for antipsychotic treatment, and intellectual disability (IQ < 70). Exclusion criteria for the HC were psychiatric diagnosis, psychiatric diagnosis in first-degree relatives, current drug abuse, and intellectual disability. All participants provided written informed consent.

### Psychopathology and cognition

Patients’ psychopathology was assessed by trained raters using the Positive and Negative Syndrome Scale (PANSS) [16]. One patient was not assessed with PANSS, resulting in 36 patients in the analyses including PANSS.

All participants were assessed with a comprehensive neurocognitive test battery by trained raters. Premorbid and current intelligence (IQ) were estimated using the Danish version of the National Adult Reading Test (DART) [17] and four subtests (vocabulary, similarities, block design, and matrix reasoning) from Wechsler Adult Intelligence Scale (WAIS-III) [18]. The Brief Assessment of Cognition in Schizophrenia (BACS) [19] was used to measure verbal fluency, working memory, verbal memory, motor skills, processing speed, and planning. Moreover, subtests from the Cambridge Neuropsychological Test Automated Battery (CANTAB) [20] were used to examine spatial span (SSP), spatial working memory (SWM), planning (Stockings of Cambridge [SOC]), mental flexibility (Intra-Extra Dimensional set shifting [IED]), sustained attention (Rapid Visual Information Processing [RVP]), and Reaction Times (RTI); see, e.g., [21–26].

#### EEG recordings

As a part of the large multimodal studies, the participants were examined with the Copenhagen Psychophysiology Test Battery [27]–[29]. After the evoked-related paradigms, participants underwent 10 min of continuous rsEEG. The rsEEG was recorded with a BioSemi ActiveTwo system (BioSemi B. V., Amsterdam, The Netherlands) with 64 active electrodes arranged according to the extended 10–20 system and with a sampling frequency of 2048 Hz. During acquisition, participants were seated in a comfortable armchair in a sound insulated cabin (40 dB). Participants were instructed to sit still, relax, and keep eyes closed. To minimize the acute and/or withdrawal effects of caffeine and nicotine, withholding from coffee intake on the test day and smoking 1 h prior to the assessment was required. A urine sample was used to screen for cannabis, cocaine, opiates, and amphetamines (Syva^®^ RapidTest d.a.u^®^ 4). Participants with positive urine screening or intake of benzodiazepines on the test day were excluded.

### EEG preparation

#### Preprocessing

The preprocessing of the raw data was carried out in Matlab (version 9.6.0.1072779 (R2019a), The MathWorks Inc., Natick, Massachusetts, USA) using the EEGLAB environment (version 2019.1) [30]. A description of the preprocessing is provided in Supplementary Material and an overview of the preprocessing steps is provided in Supplementary Figure S1.

#### Source localization

The source localization was carried out in Python (version 3.7.7) using the MNE package (version 0.19.2) [31]. First, the forward solution was computed using FreeSurfer's average head model [32]. Second, the inverse model was computed for the preprocessed data using ‘exact Low Resolution Brain Tomography’ (eLORETA) [33]. Using the inverse solution, the EEG sources were reconstructed resulting in a high-dimensional matrix. To reduce dimensionality, the source time-series were mapped to the regions of interest (ROIs) using PCA-flip, resulting in a single signal for each region. The ROIs were chosen as key regions within the DMN and defined by the CONN network parcellation based on data from the Human Connectome Project and available in the CONN toolbox [34]. The six ROIs included Medial Prefrontal Cortex (MPFC), Precuneus cortex (PCC), and Lateral Parietal (LP) cortex in each hemisphere.

#### Connectivity measures

The connectivity within the DMN was determined by the Phase Lag Index (PLI) [35], calculated using the python module dyconnmap version 1.0.2 [36]. PLI effectively eliminates volume conduction, which is a common problem when estimating functional connectivity based on EEG [35]. The data were divided into epochs of 8 s and the PLI was calculated for the first 40 epochs and averaged. The epoch duration was fixed, and the same number of epochs was used for each participant as recommended [37]. The frequency bands analyzed were delta (1.5–4 Hz), theta (4–8 Hz), alpha (8–12 Hz), and beta (12–30 Hz). The gamma band was excluded from analyses because of the risk of contamination from muscle signals [38]. The connectivity estimated by PLI is undirected; hence, the connectivity matrices are symmetric, and we therefore only consider the upper triangle. Following, we get $\frac{6\times \left(6-1\right)}{2}=15$ connectivity values per frequency band per participant.

### Modeling

**Fig. 1:**
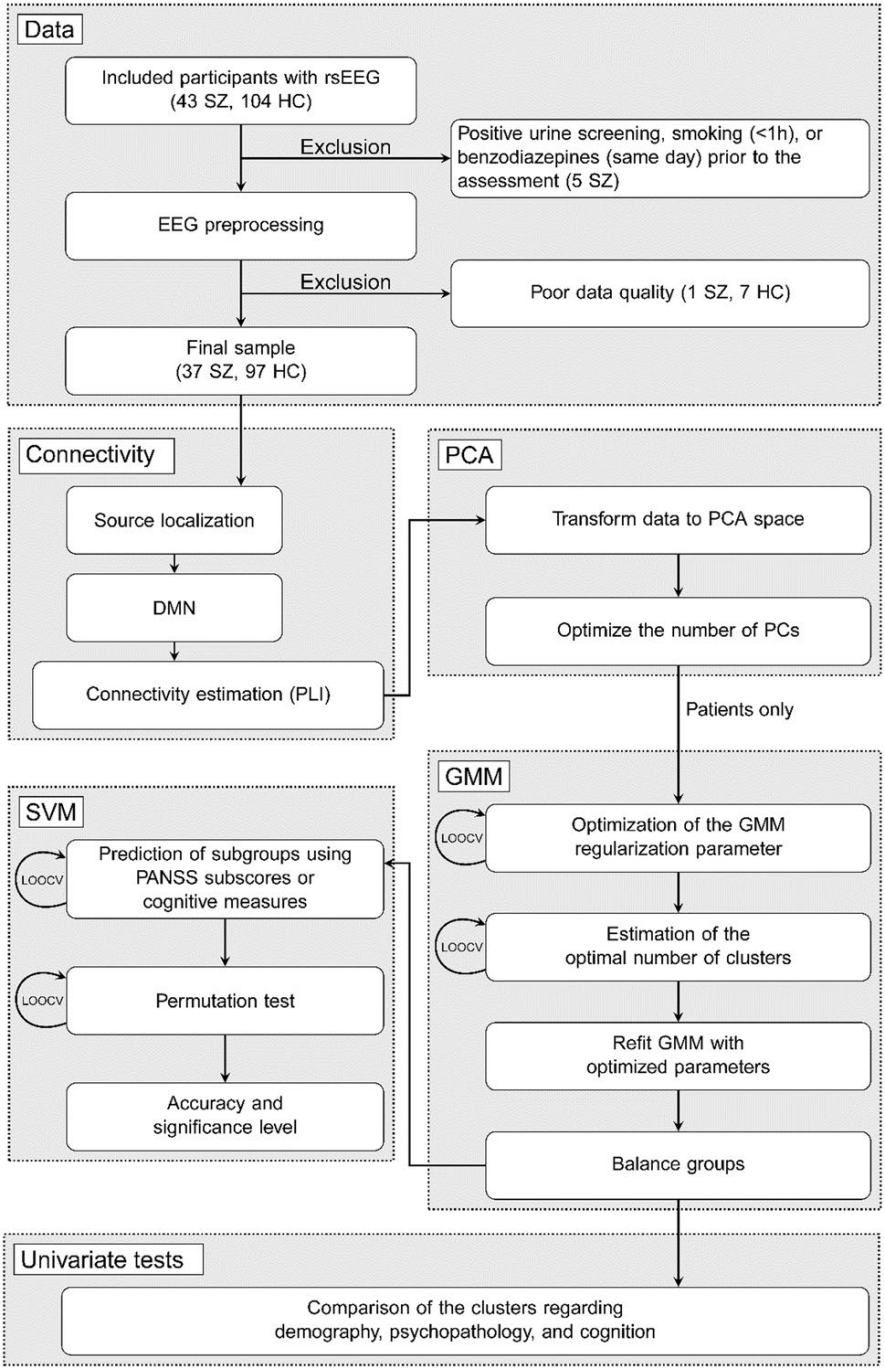
Flowchart illustrating the different steps in the analysis pipeline. *rsEEG* resting-state electroencephalography, *SZ* schizophrenia patients, *HC* healthy controls, *DMN* Default Mode Network, *PLI* Phase Lag Index, *PCA* Principal Component Analysis, *PCs* Principal Components, *SVM* Support Vector Machine, *LOOCV* Leave-one-out cross-validation, *PANSS* Positive And Negative Syndrome Scale, *GMM* Gaussian Mixture Model

All modeling was carried out in Matlab (version 9.6.0.1072779 (R2019a). The MathWorks Inc., Natick, Massachusetts). A flowchart of the study is provided in Fig. 1.

#### Principal component analysis

Principal Component Analysis (PCA) was applied to reduce the dimensionality of the feature space (15 connectivity measures per frequency band per participant). The PCA was performed on each frequency band separately. The PCA was carried out on the full data set (i.e., 37 patients and 97 HC, in total *N* = 134). The main reason for not including all frequency bands in the same analysis was the sample size-to-variable ratio. A common recommendation for PCA is that the sample size needs to be at least five times larger than the number of variables [39]. The optimal number of components to describe the data was determined by Akaike's Information Criteria (AIC) [40].

#### Gaussian mixture model

The Gaussian Mixture Model (GMM) is an unsupervised clustering algorithm that fits a selected number of Gaussian clusters to the data. As the true number of clusters in the data is unknown, the GMM was fitted to 1–8 clusters. The GMM was used to optimize subgroup attribution within the schizophrenia group based on the PCA components determined by the lowest Akaike Information Criterion (AIC) score for each frequency band separately. Because we aimed to identify subgroups of schizophrenia, only the 37 patients were used in the unsupervised clustering. There is no general agreement about the minimum sample size in cluster analyses, but Formann suggested a minimum of 2^*d*^ samples, where *d* is the number of clustering variables. To follow this recommendation, the maximum number of clustering variables in our study should be 5 [41, 42].

The regularization of the GMM was optimized and the optimal number of clusters in the data were estimated using leave-one-out cross-validation (LOOCV). To ensure robustness of our results, the GMM was repeated 50 times [43]. To be considered stable, the same number of clusters had to be the optimal in at least 90% of the runs of the GMM. Following the optimization, the GMM was refitted with the optimized regularization parameter and optimal number of clusters. For more details, please see Supplementary Material.

Group differences between the subgroups, i.e., clusters found by the GMM, were assessed using *χ*^2^-test, two-sample *t* test, or Mann–Whitney *U* test as appropriate, with respect to demography, connectivity, cognition, and psychopathology. The significance levels were corrected for multiple comparisons using the false discovery rate (FDR) [44] within each modality and considered significant for *p*_FDR_ < 0.05. Post hoc group comparisons between each of the subgroups and the HC were performed to explore the proximity of the subgroups to the HC.

#### Support vector machine

Besides assessing univariate subgroup differences, we further explored the clinical relevance of the detected subgroups, by testing if the pattern in the cognitive and psychopathological profiles, respectively, could predict subgroups. To quantify the relation between subgroup labels and patterns in cognition and psychopathology, a linear Support Vector Machine (SVM) was used to predict the frequency specific, DMN connectivity-based subgroup labels based on either PANSS sub-scores (PANSS positive, PANSS negative, and PANSS general) or cognitive measures. To avoid overfitting, only five a priori selected cognitive tests were used as predictors in the SVM. The tests were selected to cover a broad range of cognitive domains and based on subgroup differences found in our previous work [26] and included: Verbal IQ (estimated based on vocabulary and similarities from WAIS-III), verbal memory (list learning from BACS), verbal fluency (F-words from BACS), mental flexibility (IED Total errors adjusted), and reaction time (RTI five choice reaction time). The features most important for the prediction were found by inspection of the feature weights estimated by the classifier [45]. The accuracy of the SVM was estimated using LOOCV. The significance of the SVM was tested using a permutation test with 1000 permutations and the significance level was calculated by the Monte Carlo permutation *p* value [46].

## Results

Patients and HC did not differ in age, sex, and parental socioeconomic status. However, the groups differed on years of education and in use of alcohol, tobacco, opioids, benzodiazepines, stimulants, and tea (Table 1). Note that participants with acute effects of benzodiazepines on the test day were excluded from analyses. No connections differed significantly between patients and HC (Supplementary Table S3).

**Table 1:** Demographic and clinical data of the participants

	Patients	Healthy controls	
	(*N* = 37)	(*N* = 97)	
	Mean (std.)	Mean (std.)	*p* value
Cohort (PECANS 1/2)^1^	20/17	52/45	0.96
Age (years)^2^	24.4 (5.4)	24.0 (5.1)	0.62
Sex (male/female)^1^	22/15	51/46	0.47
Parental socioeconomic status (a/b/c)^1^	11/18/7	33/53/11	0.48
Education (years)^2^	12.5 (2.1)	14.4 (2.5)	** < 0.001**
Handedness score^2^	74.9 (43.2)	72.9 (52.9)	0.99
Estimated premorbid IQ^3^	20.1 (7.1)	22.6 (6.1)	0.05
Alcohol^4^	2/12/19/3/-	2/11/82/0/-	** < 0.001**
Tobacco^4^	19/4/13/0/1	45/30/16/2/1	**0.04**
Cannabis^4^	11/21/2/2/-	35/55/5/0/-	0.18
Opioids^4^	28/8/-/-/-	90/4/-/-/-	**0.004**
Stimulants^4^	27/6/2/-/-	80/14/0/-/-	0.09
Hallusinogenes^4^	28/5/-/-/-	89/4/-/-/-	0.05
Other drugs^4^	26/2/-/-/-	86/2/-/-/-	0.25
Benzodizepines^4^	21/12/1/-/-	92/1/0/-/-	** < 0.001**
Coffee (no/yes)^1^	17/20	36/60	0.37
Tea (no/yes)^1^	12/25	51/46	**0.04**
Other caffeine (no/yes)^1^	16/20	37/58	0.57
CGI severity	4.1 (0.6)	–	–
GAF symptom	38.8 (9.1)	–	–
GAF function	45.9 (12.7)	–	–
SOFAS	41.7 (11.7)	–	–
PANSS positive	18.2 (4.3)	–	–
PANSS negative	18.1 (7.4)	–	–
PANSS general	36.8 (9.7)	–	–
PANSS total	73.0 (17.8)	–	–
DUP (days)	93.1 (124.4)	–	–
DUI (days)	50.1 (68.3)		–

Substance use are categorized between 0 and 4 (0 = never tried, 1 = tried few times, 2 = regular use, 3 = harmful use, and 4 = dependency). Signif*icant group differences in bold*

Estimated premorbid IQ is based on the Danish Adult Reading Test (DART) [17]. Handedness is estimated with The Edinburgh Handedness Inventory [47]. Duration of untreated illness (DUI) was defined as the time from initial decline in functioning due to psychosis-related symptoms and duration of untreated psychosis (DUP) was defined as the time from the first continuous psychotic symptom [48]

*CGI* Clinical global impression, *GAF* Global assessment of functioning, *SOFAS* Social and occupational functioning assessment scale [49], *PANSS* Positive and negative syndrome scale [16]

^1^χ^2^ test

^2^Mann–Whitney *U* test

^3^Two-sample *t* test

^4^Fisher’s exact test

We performed four PCAs, one for each frequency band, each with 15 features (6 ROIs resulting in $\frac{6\times \left(6-1\right)}{2}=15$ undirected connections). For the delta, alpha, and beta bands, the optimal number of PCs was four, and for the theta band, the optimal number of PCs was five based on the AIC score. For details on performance, the AIC curves are provided in Supplementary Figure S2. The performances of the GMMs across different number of clusters are provided in Supplementary Figure S3. The delta and alpha frequency bands revealed no patient clusters, as only one cluster was found to be optimal in 44% and 100% of the runs, respectively. However, using the DMN connectivity, we identified two statistically significant clusters in the theta band, and two statistically significant clusters in the beta band, which were found to be optimal in 98% and 100% of the runs, respectively. Because the distribution of patients in the theta and beta band subgroups was unbalanced, patients were assigned to the clusters based on the posterior probability of the GMM.

### Theta subgroups

#### Connectivity

**Fig. 2:**
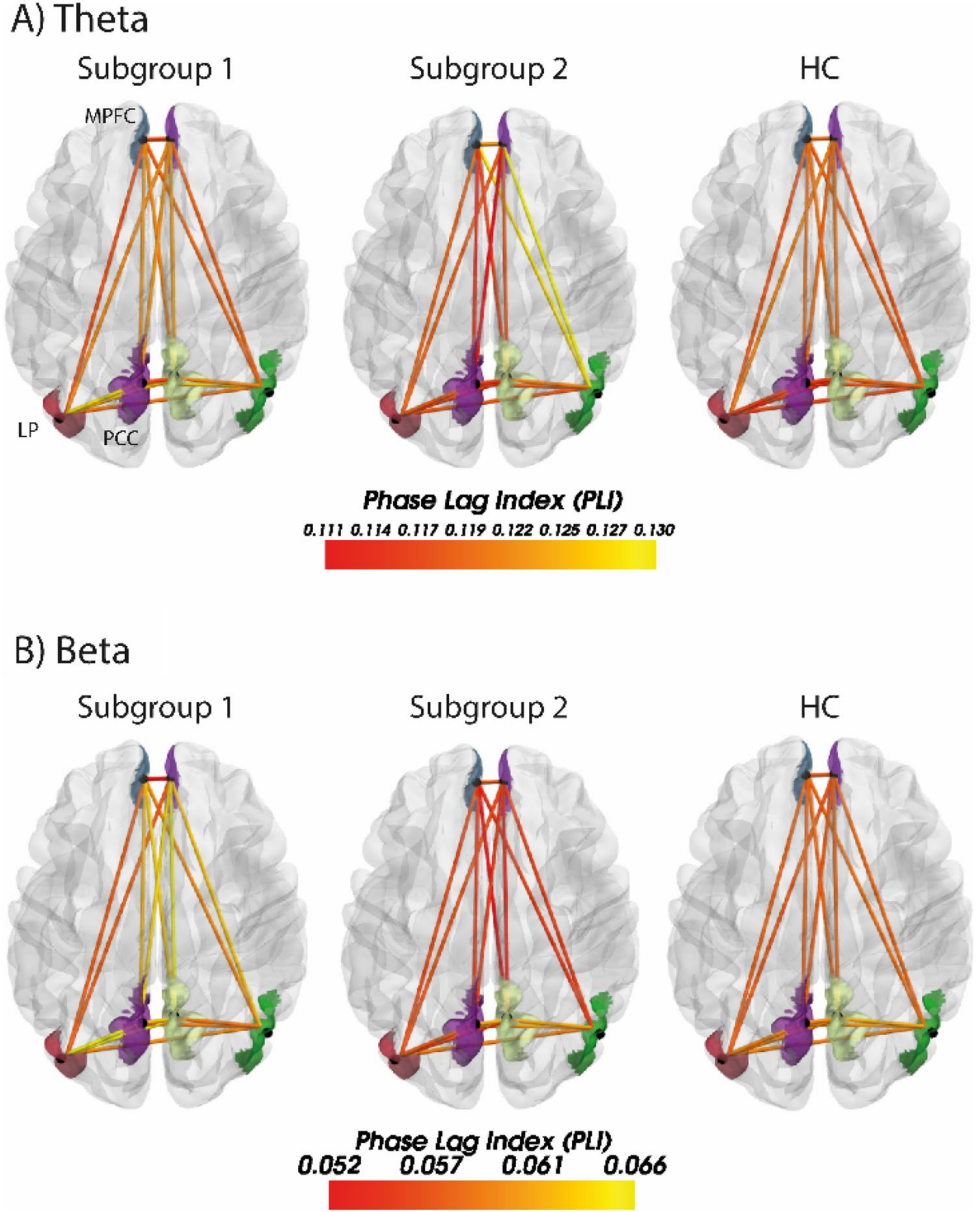
Top view of the regions of the Default Mode Network from the CONN network parcellation: Medial prefrontal cortex (MPFC), Precuneus Cortex (PCC), and Lateral Parietal (LP). **A** Theta subgroups and healthy controls (HC) and **B** the Beta subgroups and HC. The connection strengths are weighted by the Phase Lag Index (PLI)

The loadings of the five PCs used as predictors in the GMM are provided in Supplementary Figure S4. The two ‘theta subgroups’ did not differ significantly on any connection; see Fig. 2 and Table 2.

**Table 2:** Connectivity estimated with Phase Lag Index and cognitive scores for the theta- and beta subgroups and the HC

	Theta				Beta			
	SZ1	SZ2	HC	SZ1 vs SZ2	SZ1	SZ2	HC	SZ1 vs SZ2
	(*N* = 19)	(*N* = 18)	(*N* = 97)	*p *value	(*N* = 19)	(*N* = 18)	(*N* = 97)	*p* value
Functional connectivity								
L LP vs L MPFC	0.116	0.118	0.117	0.701	0.057	0.056	0.057	0.667
L LP vs L PCC	0.128	0.118	0.116	**0.025**	0.066	0.057	0.059	0.003*
L LP vs R LP	0.120	0.117	0.116	0.619	0.060	0.059	0.059	0.760
L LP vs R MPFC	0.121	0.118	0.120	0.796	0.057	0.056	0.057	0.790
L LP vs R PCC	0.125	0.116	0.115	**0.026**	0.065	0.056	0.059	<0.001*
L MPFC vs L PCC	0.122	0.113	0.119	**0.036**	0.062	0.054	0.057	0.010*
L MPFC vs R LP	0.118	0.127	0.118	**0.019**	0.059	0.055	0.058	**0.048**
L MPFC vs R MPFC	0.117	0.123	0.120	0.309	0.053	0.057	0.057	**0.046**
L MPFC vs R PCC	0.121	0.115	0.119	0.190	0.063	0.052	0.057	<0.001*
L PCC vs R LP	0.126	0.117	0.116	0.139	0.059	0.060	0.060	0.443
L PCC vs R MPFC	0.122	0.111	0.119	**0.016**	0.064	0.055	0.057	0.001*
L PCC vs R PCC	0.117	0.115	0.115	0.781	0.062	0.061	0.060	0.783
R LP vs R MPFC	0.118	0.130	0.117	**0.008**	0.062	0.055	0.057	0.008*
R LP vs R PCC	0.120	0.120	0.118	0.959	0.058	0.060	0.061	0.311
R MPFC vs R PCC	0.123	0.117	0.119	0.208	0.065	0.053	0.057	<0.001*
Cognition								
Estimated premorbid IQ	18.9	21.2	22.6	0.365	18.6	21.7	22.6	0.211
Verbal IQ	97.7	103.6	112.6	0.326	99.8	101.6	112.6	0.760
Performance IQ	101.2	104.5	108.6	0.616	103.6	102.0	108.6	0.690
Fullscale IQ	99.3	104.4	111.9	0.349	101.6	102.2	111.9	0.909
List learning	55.2	56.4	58.2	0.615	57.4	54.1	58.2	0.170
Number sequence	22.6	22.2	23.0	0.704	22.5	22.3	23.0	0.871
TOKEN	67.2	65.8	73.0	0.795	62.9	69.9	73.0	0.097
Fluency Supermarked	26.9	25.7	32.8	0.635	26.1	26.5	32.8	0.860
Fluency F	13.2	14.2	16.8	0.533	13.7	13.8	16.8	0.921
Fluency S	15.1	16.2	18.0	0.501	16.1	15.2	18.0	0.804
Symbol coding	55.0	58.9	67.3	0.322	58.2	55.8	67.3	0.539
Tower of London	19.8	18.7	19.7	0.055	19.9	18.7	19.7	0.119
Spatial span	7.5	7.2	7.7	0.669	7.8	6.9	7.7	0.087
SWM Strategy	27.5	27.8	23.8	0.868	25.9	29.5	23.8	0.119
SWM Totalerrors	10.3	9.4	5.6	0.960	9.4	10.4	5.6	0.496
SOC Problems solved min moves	10.5	10.1	10.3	0.467	10.2	10.4	10.3	1.000
SOC initialthinking	13429.1	13293.3	11128.8	0.766	12379.5	14396.6	11128.8	0.692
IED Stages completed	8.1	8.7	8.8	**0.047**	8.6	8.2	8.8	0.216
IED Total errors adjusted	31.1	19.9	16.1	0.679	19.4	31.5	16.1	0.247
IED EDS errors	13.2	7.8	6.2	0.292	6.7	14.4	6.2	**0.022**
RTI simple reactiontime	305.5	313.6	293.2	0.293	286.2	332.9	293.2	**0.029**
RTI simple movementtime	389.9	420.6	388.1	0.502	381.5	428.9	388.1	0.088
RTI choice reactiontime	343.2	330.3	318.9	0.431	322.4	351.1	318.9	0.095
RTI choice movementtime	386.4	389.1	371.5	0.642	374.6	400.9	371.5	0.440
RVP A'	0.9	0.9	1.0	0.418	0.9	1.0	1.0	0.398

Group comparisons of the subgroups for the theta and beta band, respectively, are performed using two-sample *t* test or Mann–Whitney *U* test as appropriate. Significant differences are in bold and differences surviving false discovery rate adjustment are marked with an asterisk

Estimated premorbid IQ is based on the Danish Adult Reading Test (DART)

*SZ1* Subgroup 1, *SZ2* Subgroup 2, *HC* Healthy controls, *L* Left, *R* Right, *LP *Lateral parietal, MPFC Medial prefrontal cortex, *PCC* Precuneus cortex, *SWM* Spatial working memory, *SOC* Stockings of cambridge, *IED* Intra-extra dimensional set shifting, *RTI* Reaction times, *RVP* Rapid visual Information processing

Post hoc tests (Supplementary Tables S4 and S5) revealed that ‘Theta Subgroup 1’ had increased connectivity compared to HC between left Lateral Parietal and left Precuneus Cortex (*p*_FDR_ = 0.021) and left Lateral Parietal and right Precuneus Cortex (*p*_FDR_ = 0.033) and ‘Theta Subgroup 2’ had increased connectivity between right Lateral Parietal and right Medial Prefrontal Cortex (*p*_FDR_ = 0.007).

#### Subgroup differences in clinical and cognitive measures

**Fig. 3:**
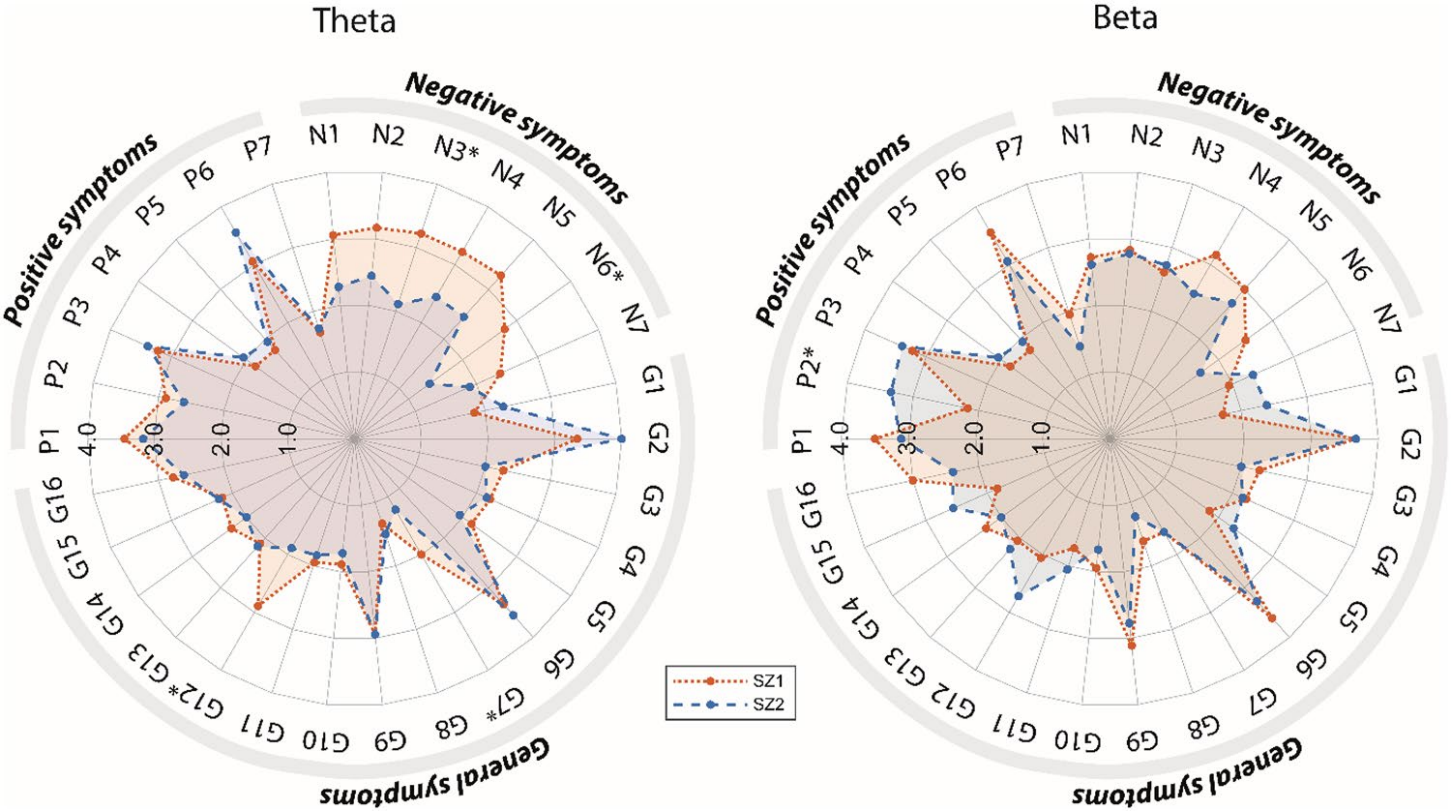
Psychopathological (PANSS) profiles of the schizophrenia subgroups, subgroup 1 (SZ1, red dotted line) and subgroup 2 (SZ2, blue dashed line), derived using the theta frequency band (left) and the beta frequency band (right). Significant differences (*p* < 0.05, uncorrected) between the subgroups are indicated with an asterisk. See text and Supplementary Tables S1 and S2 for details

The two theta subgroups did not differ on demography, substance use, or clinical measures. However, Theta Subgroup 1 tended to show poorer functioning and more profound negative symptoms than Theta Subgroup 2 [GAF symptom (*p* = 0.049), GAF function (*p* = 0.01), and PANSS negative sub-score (*p* = 0.01)]. The psychopathological profiles of the theta subgroups are illustrated in Fig. 3 (left) and comparison of the individual PANSS items is provided in Supplementary Table S1. Group comparison revealed no differences in cognitive performance between the theta subgroups (see Supplementary Figure S7 for visualization of profiles). Both subgroups showed cognitive deficits compared to HC (Table 2 and Supplementary Table S4).

#### Predictive clinical profiles

Even though no univariate significant differences in psychopathology or cognitive performance exist between the identified subgroups, the patterns in the cognitive and psychopathological profiles may be predictive of the subgroups. To explore the predictive power of the cognitive and psychopathological profiles between the subgroups, the a priori selected cognitive variables (verbal IQ, verbal memory, verbal fluency, mental flexibility, and reaction time), and the PANSS sub-scores (PANSS positive, PANSS negative, and PANSS general) were used as predictors in a SVM, respectively. Using the cognitive variables, the theta subgroups were not predicted better than chance. However, using the PANSS sub-scores, the theta subgroups were predicted with an accuracy of 69.4% (*p* = 0.003). Based on the coefficient weights of the SVM (Supplementary Figure S6), we found that the prediction was primarily driven by negative symptoms. However, the positive symptoms did also contribute to the prediction, but in the opposite direction, indicating that Theta Subgroup 1 had a pattern of more negative symptoms and less positive symptoms compared to Theta Subgroup 2.

### Beta subgroups

#### Beta connectivity

The loadings of the four PCs used as predictor variables in the GMM are provided in Supplementary Figure S5. Comparison of the two beta subgroups showed significantly higher connectivity in several connections in Subgroup 1 compared to Subgroup 2 (Fig. 2 and Table 2). Group comparison of each of the beta subgroups to the HC revealed increased connectivity in Subgroup 1 in several connections, but no significant differences in Subgroup 2 (Supplementary Tables S6 and S7).

#### Subgroup differences in clinical and cognitive measures

The two beta subgroups did not differ on demography, substance use, or any clinical measures. The psychopathological profiles of the beta subgroups are illustrated in Fig. 3 (right) and comparison of the individual PANSS items is provided in Supplementary Table S2. The beta subgroups did not differ significantly on any cognitive measures (Table 2) and both subgroups had poorer performance than HC in several cognitive domains (Supplementary Tables S6 and S7). For visualization of the cognitive profiles, please see Supplementary Figure S7.

#### Predictive clinical profiles

To explore whether the patterns within cognition or psychopathology were predictive of the beta subgroups, again SVM was applied. The subgroups based on beta band connectivity were significantly predicted by the SVM using the five cognitive measures with an accuracy of 63.6% (*p* = 0.034). The prediction was driven by mental flexibility, verbal IQ, and the measure of reaction time. For details on coefficient weights, see Supplementary Figure S6. Beta Subgroup 2 made more errors on IED (poorer mental flexibility) and showed longer reaction times, but higher verbal IQ. Using the PANSS sub-scores as predictors, the beta subgroups were not predicted better than chance.

## Discussion

Using the functional connectivity in the theta and beta frequency band, respectively, our data-driven approach revealed that patients could be separated into two subgroups that did not differ on any demographic measures. The functional connectivity in the delta and alpha frequency bands did not support a subdivision of the schizophrenia patients using the current approach.

The identified theta subgroups did not differ in connectivity, but Theta Subgroup 1 tended to have poorer functioning and more negative symptoms. Furthermore, the patterns in the psychopathology significantly predicted the theta subgroups, primarily driven by the negative symptoms. Interestingly, previous data have also observed differences in theta power between subgroups of patients characterized by positive and negative symptoms, respectively [50–52]. The psychopathological profile in theta subgroup 1 may indicate that this subgroup include patients with more severe and persistent negative symptoms, and thus be an early biological indicator of what will later be categorized as “deficit schizophrenia” [53, 54].

The two identified subgroups in the beta band showed increased and decreased connectivity respectively between key regions of the DMN, compared to HC. Notably, the alterations in the beta connectivity could not be detected in the patient group before subgrouping as the alterations had opposite directionality in the two beta subgroups, hence supporting the notion of subgroups within schizophrenia patients. One study divided the patients into subgroups based on psychopathology and showed that patients with more negative symptoms had greater beta power in the left hemisphere regions compared to those with fewer negative symptoms. They also showed that beta power was positively correlated with negative symptoms [55]. We did not find any differences in psychopathology between the two beta subgroups, but together these results indicate that the directionality of the beta oscillation alterations is different between subgroups of patients with schizophrenia. The identified beta subgroups were significantly predicted by the five a priori selected cognitive variables, suggesting a coupling between measures of externally directed cognition and subgroups of patients with schizophrenia defined by within-network connectivity in the DMN. Accordingly, beta oscillations have previously been associated with emotional and cognitive processes [56] and spontaneous cognitive operations during conscious rest in healthy participants [57]. While the cognitive variables in question are directly relevant for solving externally directed tasks, it is unclear how these variables are associated with the function of DMN sub-networks normally associated with internally directed cognition.

To the best of knowledge, no previous study has aimed at defining subgroups of antipsychotic-naïve first-episode patients with schizophrenia based on functional connectivity measures using rsEEG. Only one study using rsfMRI has had this focus [58], where two subgroups of patients were identified with decreased and increased connectivity, respectively, within a triple-network system including the DMN. Moreover, these subgroups were associated with specific cognitive deficits [58]. Although this rsfMRI study focused on DMN connectivity in a triple-network model and we used a within-network connectivity measure, the finding of subgroups defined by DMN connectivity and the association with cognitive scores appears to be robust across different temporal resolutions applied in rsfMRI and rsEEG.

Another line of research has investigated the classification accuracy of discriminating between clinically defined subgroups of antipsychotic-naïve patients with schizophrenia based on functional connectivity measures. Two of the earlier studies using rsfMRI tested the discriminating power in classifying patients with and without auditory verbal hallucinations (AVH). They reported an accuracy of 75.6% (*p* < 0.01) and found that the most discriminative connections were associated with the DMN [59]. Importantly, these studies investigated the discriminative power of functional connectivity measures between clinically defined subgroups, which may not capture the underlying biological mechanisms of schizophrenia [60]. Indeed, the high heterogeneity within schizophrenia may be due to biologically different subgroups within the diagnostic category of schizophrenia. Hence, effective treatment may be blurred by different biological disturbances within each subgroup and the identification of biological distinct subgroups may therefore help in the development of treatment targeting the specific biological disturbances [26].

The use of data-driven methods, e.g., clustering, enables detection of subtle biological differences and hence allows subgrouping based on patterns not visible to the naked eye. A limitation of cluster analysis is that no ground truth is available, and thus, the subgroups may reflect irrelevant traits rather than clinically relevant traits such as treatment response. To confirm the relevance of the subgroups, we therefore characterized the subgroups regarding psychopathology and cognition as suggested by Marquand et al. [61]. In line with our previous study [26], the identified subgroups were not clinically separable using univariate tests, yet the patterns within cognitive- or psychopathological measures significantly predicted the subgroups, emphasizing the importance of data-driven methods.

Taken together, aberrations in DMN connectivity appear associated with schizophrenia around the onset of manifest symptoms and resting-state paradigms provide some evidence of the existence of subgroups in patients with schizophrenia defined by DMN connectivity. It should, however, be noted that multiple approaches for analyzing EEG data exist, and to the best of our knowledge, there is no consensus on which one is the best regarding functional connectivity estimation [62, 63]. The optimal approach may very well depend on the data and the specific hypothesis in question [14]. Standardization of approaches and reports may lead to easier comparison between studies as suggested in a recent review [64]. Despite these limitations, the present study demonstrates for the first time the feasibility of using functional connectivity of the DMN measured with rsEEG to define valid neurophysiological subgroups of antipsychotic-naïve first-episode patients with schizophrenia.

The patients included in the current study were strictly antipsychotic-naïve and first-episode patients, and hence, effects of chronicity and previous exposure to antipsychotic medication can be ruled out. The sample is, however, relatively small in terms of subgrouping and may limit the ability to detect differences between subgroups and associations between subgroups and cognitive/clinical measures due to limited power. Optimally, our finding should be replicated in a larger, independent sample, but as an independent sample was not available, we estimated the cluster reproducibility using cross-validation and confirmed the relevance of the identified subgroups by biological measures, i.e., psychopathology and cognition.

### Conclusions

Functional connectivity measures in the DMN provides a novel, data-driven means to stratify patients according to differences in brain networks, suggesting subgroups of patients with different underlying pathology. We showed that such subgroups exists and is clinically relevant in terms of cognitive function and negative symptoms. The results of the current study support the notion of biological subgroups of schizophrenia and endorse the application of data-driven methods to recognize the patterns within the subtle differences among schizophrenia patients.

### Supplementary Information

Below is the link to the electronic supplementary material.

Supplementary file1 (DOCX 6643 KB)

## Data Availability

The data that support the findings of this study are available upon reasonable request from the corresponding author, KSA. The data are not publicly available due to privacy of research participants.
