# Supplementary material for: Clustering of antipsychotic-naïve patients with schizophrenia based on functional connectivity from resting-state electroencephalography
Source: Eur Arch Psychiatry Clin Neurosci. 2023 Feb 2;273(8):1785–96. doi: 10.1007/s00406-023-01550-9 (PMC10713774; doi:10.1007/s00406-023-01550-9)
Supplement: Supplementary file 1 — Supplementary file1 (DOCX 6643 KB) [file 406_2023_1550_MOESM1_ESM.docx]

Clustering of antipsychotic-naïve patients with schizophrenia based on functional connectivity from resting-state electroencephalography

**Authors:**

Karen S. Ambrosen^1^*, Fanny Fredriksson^1^*, Simon Anhøj^1^, Nikolaj Bak^2^, Edwin van Dellen^3^, Livia Dominicus^3^, Cecilie K. Lemvigh^1^, Mikkel E. Sørensen^1^, Mette Ø. Nielsen^1,4^, Kirsten B. Bojesen^1^, Birgitte Fagerlund^1,5^, Birte Y. Glenthøj^1,4^, Bob Oranje^1^, Lars K. Hansen^6^, and Bjørn H. Ebdrup^1,4^.

**Affiliations:**

^1^Center for Neuropsychiatric Schizophrenia Research (CNSR) and Center for Clinical Intervention and Neuropsychiatric Schizophrenia Research (CINS), Copenhagen University Hospital - Mental Health Services CPH, Glostrup, Denmark

^2^H. Lundbeck A/S, Valby, Denmark

^3^Department of Psychiatry, Brain Center Rudolf Magnus, University Medical Center Utrecht, Utrecht, The Netherlands

^4^Department of Clinical Medicine, Faculty of Health and Medical Sciences, University of Copenhagen, Copenhagen, Denmark

^5^Department of Psychology, University of Copenhagen, Denmark

^6^DTU Compute, Department of Applied Mathematics and Computer Science, Technical University of Denmark, Kgs. Lyngby, Denmark

* The authors contributed equally

**Corresponding author:** Karen S. Ambrosen, Nordstjernevej 41, 2600 Glostrup, Denmark, Tel. +45 28439264. Email: [karen.marie.sandoe.ambrosen@regionh.dk](mailto:karen.marie.sandoe.ambrosen@regionh.dk), ORCID: 0000-0002-5638-2357.

# Supplementary Material

## Preprocessing

The preprocessing of the raw data was carried out in Matlab (version 9.6.0.1072779 (R2019a), The MathWorks Inc., Natick, Massachusetts, USA) using the EEGLAB environment (version 2019.1) [1]. The data was down-sampled to 256 Hz for faster computation and band-pass filtered between 1 and 100 Hz using a Hamming windowed-sinc FIR-filter. Flat lining signals, noisy channels, bursts, and events of abnormally high power were removed and interpolated by artifact subspace reconstruction (ASR) using the EEGLAB plugin *clean_artifacts* [2]. The signal was average referenced, and notch filtered between 48-51 Hz using a Hamming windowed-sinc FIR-filter to remove power line noise. Lastly, Independent Component Analysis (ICA) decomposition was carried out using the infomax ICA algorithm, with the natural gradient feature and extended-ICA algorithm. The components were analyzed with *IClabel* [3], and the components, where the brain class had higher probability than the other classes (muscle, eye, heart, line noise, channel noise, or other) were used in the reconstruction. A final visual check of the data was conducted, and subjects were excluded if the preprocessing had not performed satisfactorily according to visual inspection. An overview of the preprocessing steps is provided in Supplementary Figure S1.

## Gaussian Mixture Model

The Gaussian Mixture Model (GMM) is an unsupervised clustering algorithm that fits a selected number of Gaussian clusters to the data. The regularization of the GMM was optimized using leave-one-out cross-validation (LOOCV). The regularization parameter achieving the lowest negative log likelihood (averaged across the cross-validation left-out-samples for each cluster configuration) was chosen as the optimal parameter. Second, the optimal number of clusters in the data was estimated. The GMM is non-deterministic due to the incorporation of the Expectation-Maximization algorithm initialized with the K-means++ algorithm. To ensure robustness of our results, the GMM incorporating LOOCV was repeated 50 times with the optimized regularization parameter [4]. The negative log likelihood was averaged across the left-out-samples for the different number of clusters. The number of clusters with the lowest mean negative log likelihood was selected. To be considered stable, the optimal number of clusters had to be the optimal in at least 90% of the runs of the GMM. Following the optimization, the GMM was refitted with the optimized regularization parameter and optimal number of clusters.

## References

[1] A. Delorme and S. Makeig, “EEGLAB: an open source toolbox for analysis of single-trial EEG dynamics including independent component analysis,” *J. Neurosci. Methods*, vol. 134, pp. 9–21, 2004.

[2] T. R. Mullen *et al.*, “Real-time neuroimaging and cognitive monitoring using wearable dry EEG,” *IEEE Trans. Biomed. Eng.*, vol. 62, no. 11, pp. 2553–2567, 2015, doi: 10.1109/TBME.2015.2481482.

[3] L. Pion-Tonachini, K. Kreutz-Delgado, and S. Makeig, “ICLabel: An automated electroencephalographic independent component classifier, dataset, and website,” *Neuroimage*, vol. 198, pp. 181–197, 2019.

[4] G. Varoquaux, P. R. Raamana, D. A. Engemann, A. Hoyos-Idrobo, Y. Schwartz, and B. Thirion, “Assessing and tuning brain decoders: Cross-validation, caveats, and guidelines,” *Neuroimage*, vol. 145, no. August 2015, pp. 166–179, 2017, doi: 10.1016/j.neuroimage.2016.10.038.

# Supplementary Figures


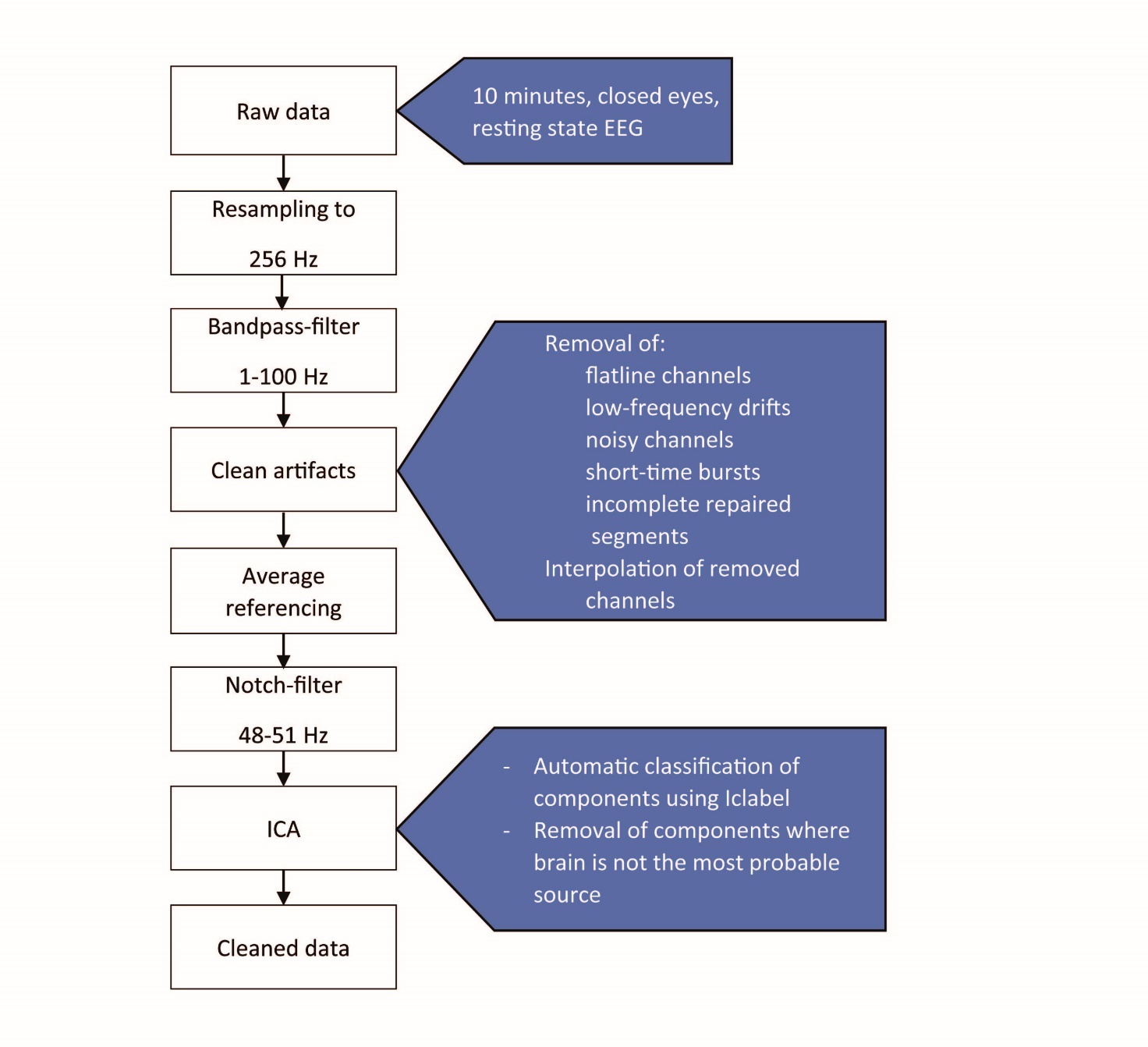


Supplementary Figure S1: EEG Preprocessing pipeline


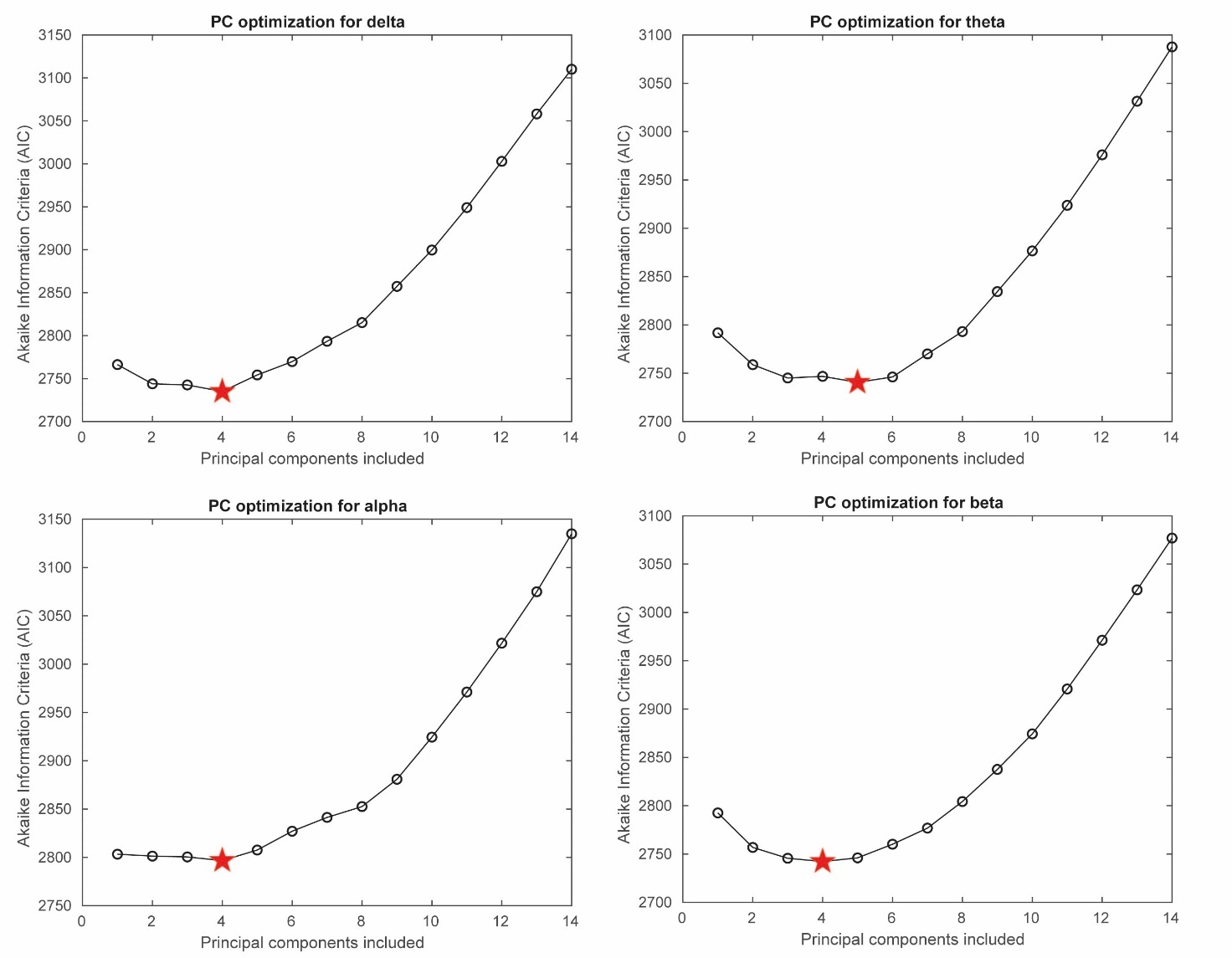


Supplementary Figure S2: Akaike Information Criteria (AIC) as function of number of principal components (PCs) included for the four frequency bands. The red stars mark the optimal number of PCs.


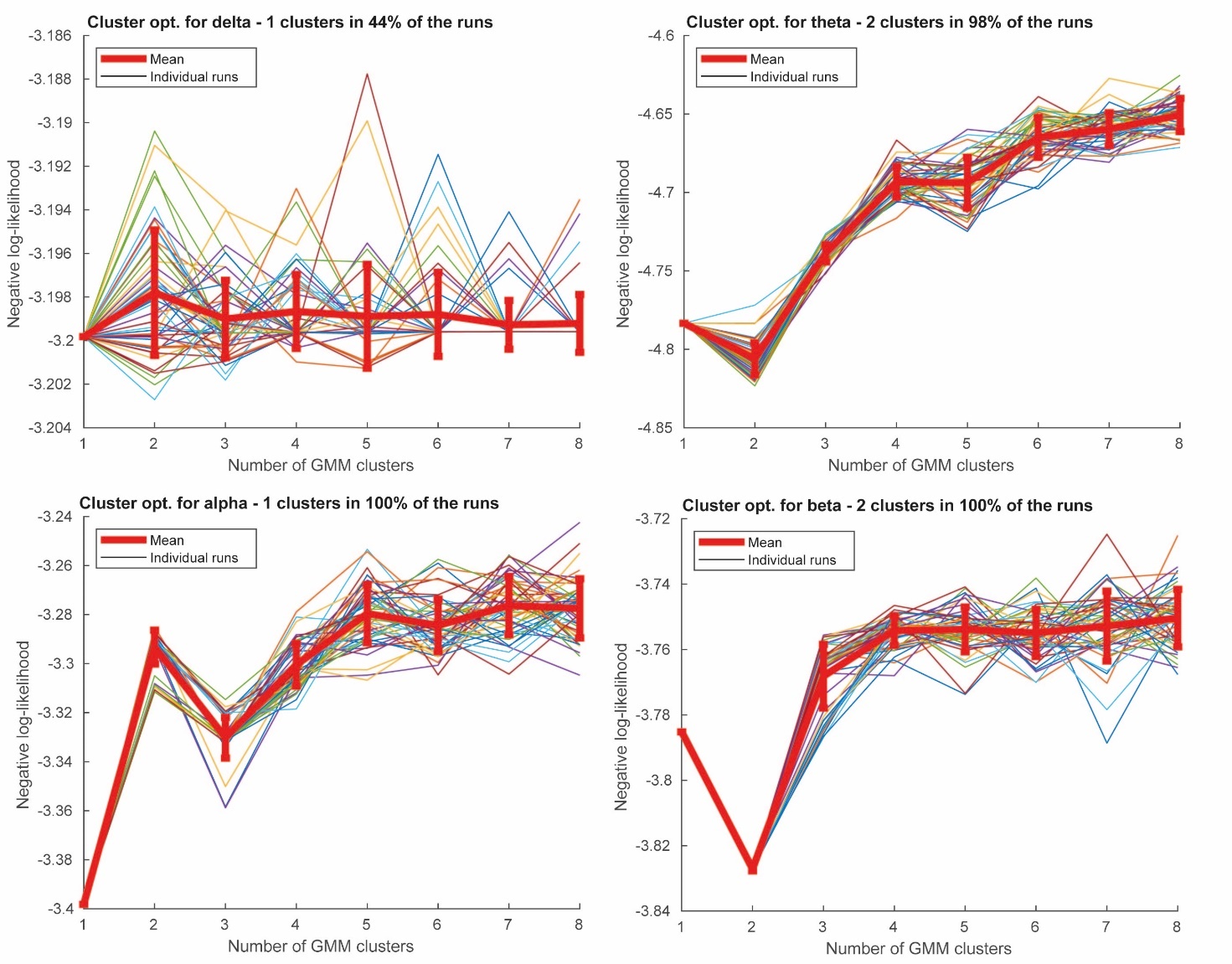


Supplementary Figure S3: Negative log-likelihood as function of the number of clusters when running the GMM. The thin lines indicate the 50 separate restarts of the GMM, and the thick red lines mark the mean and standard deviation of the restarts.


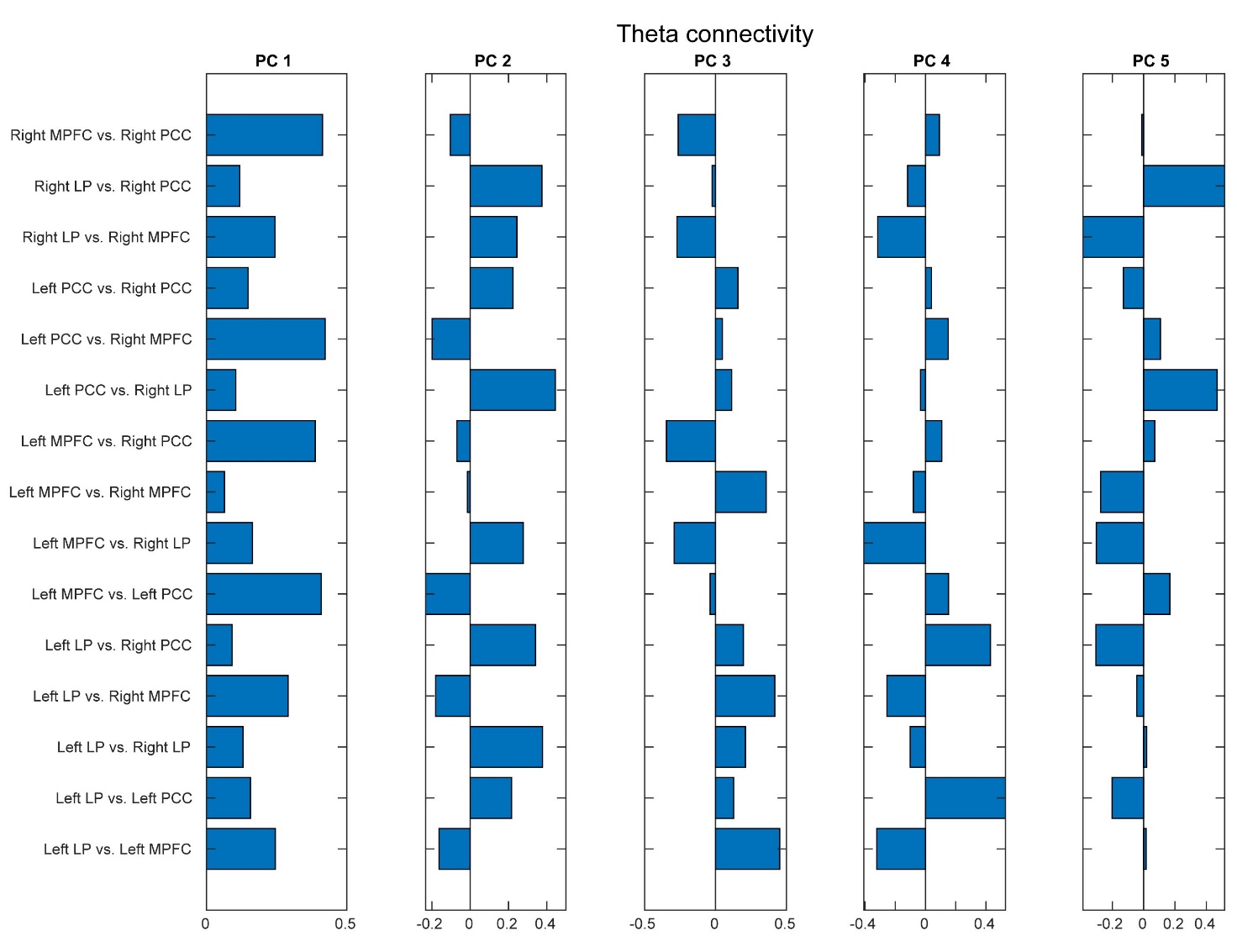


Supplementary Figure S4: Loadings of the five principal components (PCs) found to be optimal when analyzing the functional connectivity within the theta frequency band. The first PC loaded primarily on connections between Medial Prefrontal Cortex (MPFC) and Precuneus Cortex (PCC), both within and between hemispheres. PC 2 loaded primarily on connections between Lateral Parietal (LP) and PCC. PC 3 loaded primarily on connections involving MPFC, PC 4 on connections involving LP, and PC 5 on connections involving LP, especially right LP.


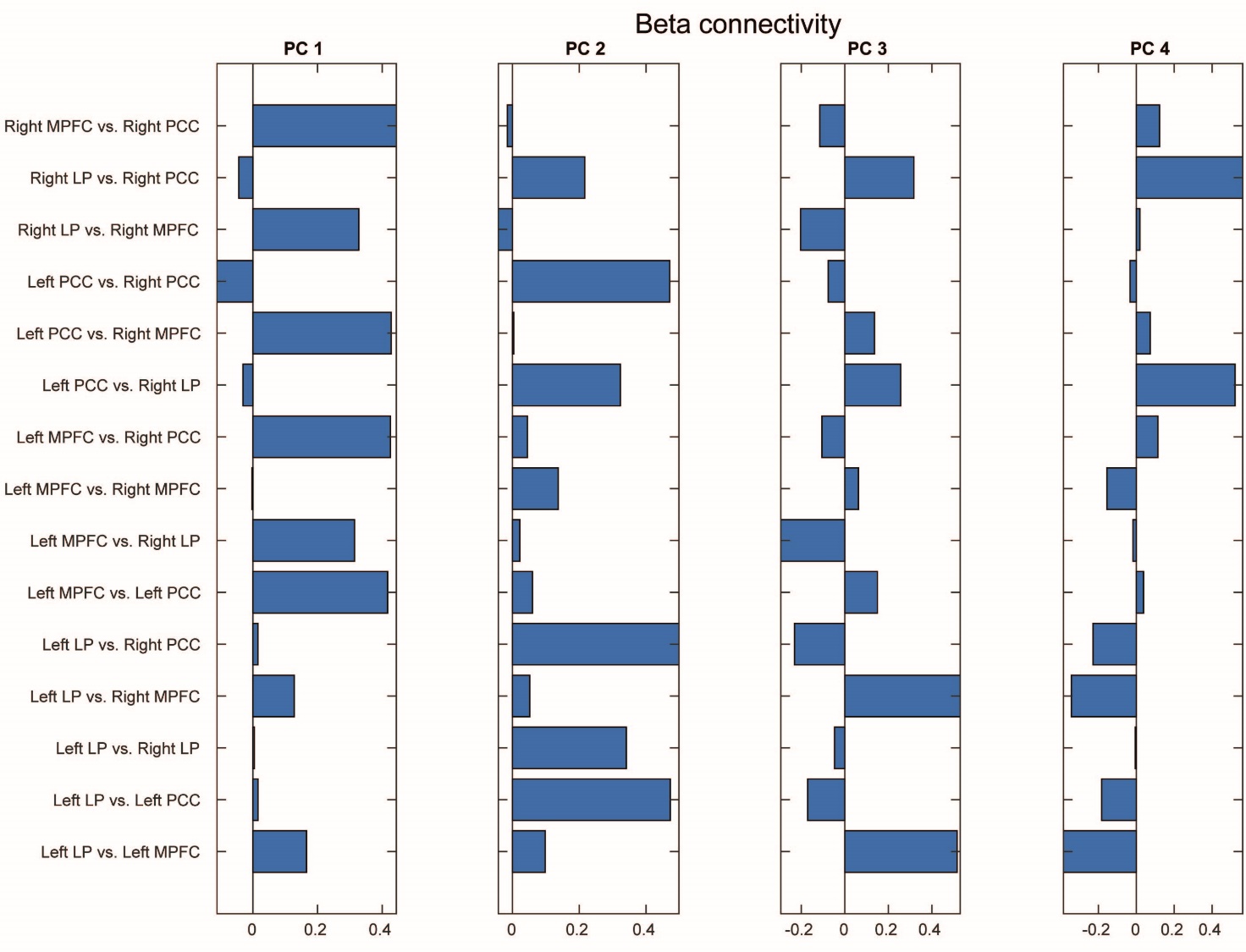


Supplementary Figure S5: Loadings of the four principal components (PCs) found to be optimal when analyzing the functional connectivity within the beta frequency band. The first PC loaded primarily on connections involving Medial Prefrontal Cortex (MPFC). PC 2 loaded primarily on connections not involving MPFC, i.e., all connections within and between Lateral Parietal (LP) and Precuneus Cortex (PCC) bilaterally. PC 3 loaded primarily on connections between left LP and left/right MPFC, and PC 4 loaded primarily on connections between right LP and left/right PCC.


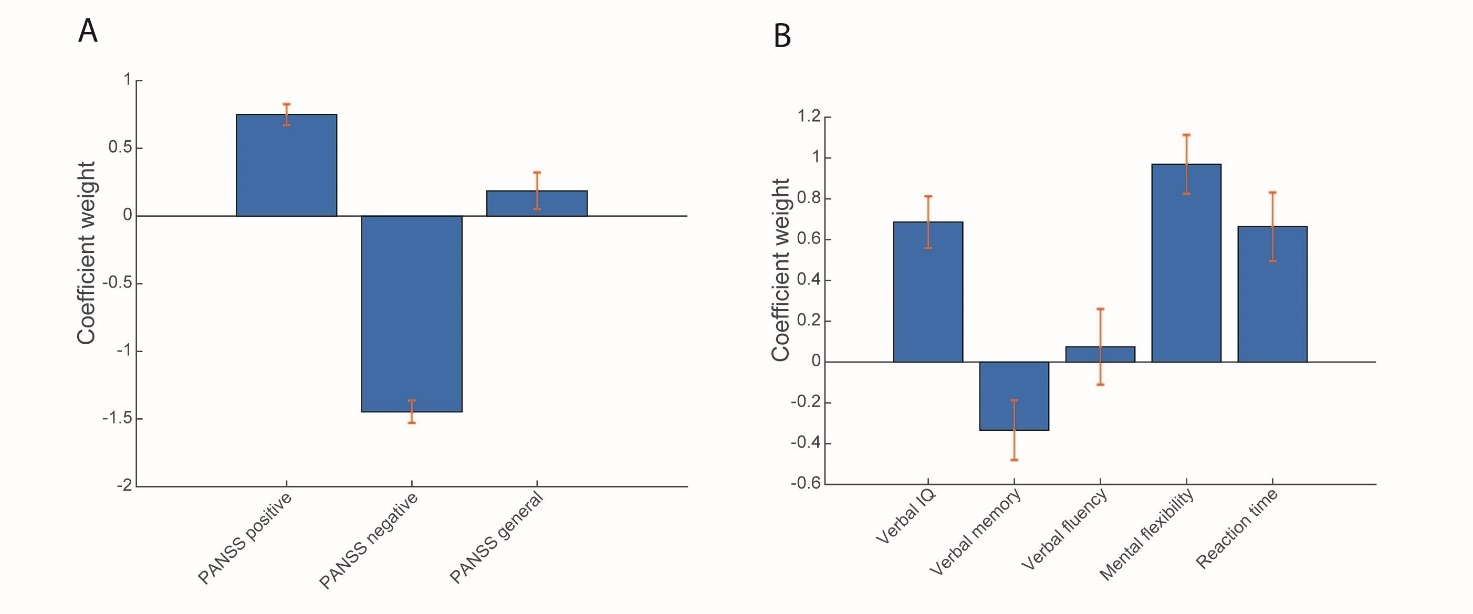


Supplementary Figure S6: Coefficient weights of the Support Vector Machine predicting the subgroup labels of the A) Theta subgroups using the psychopathology subscores as predictors, and B) Beta subgroups using five cognitive scores as predictors.


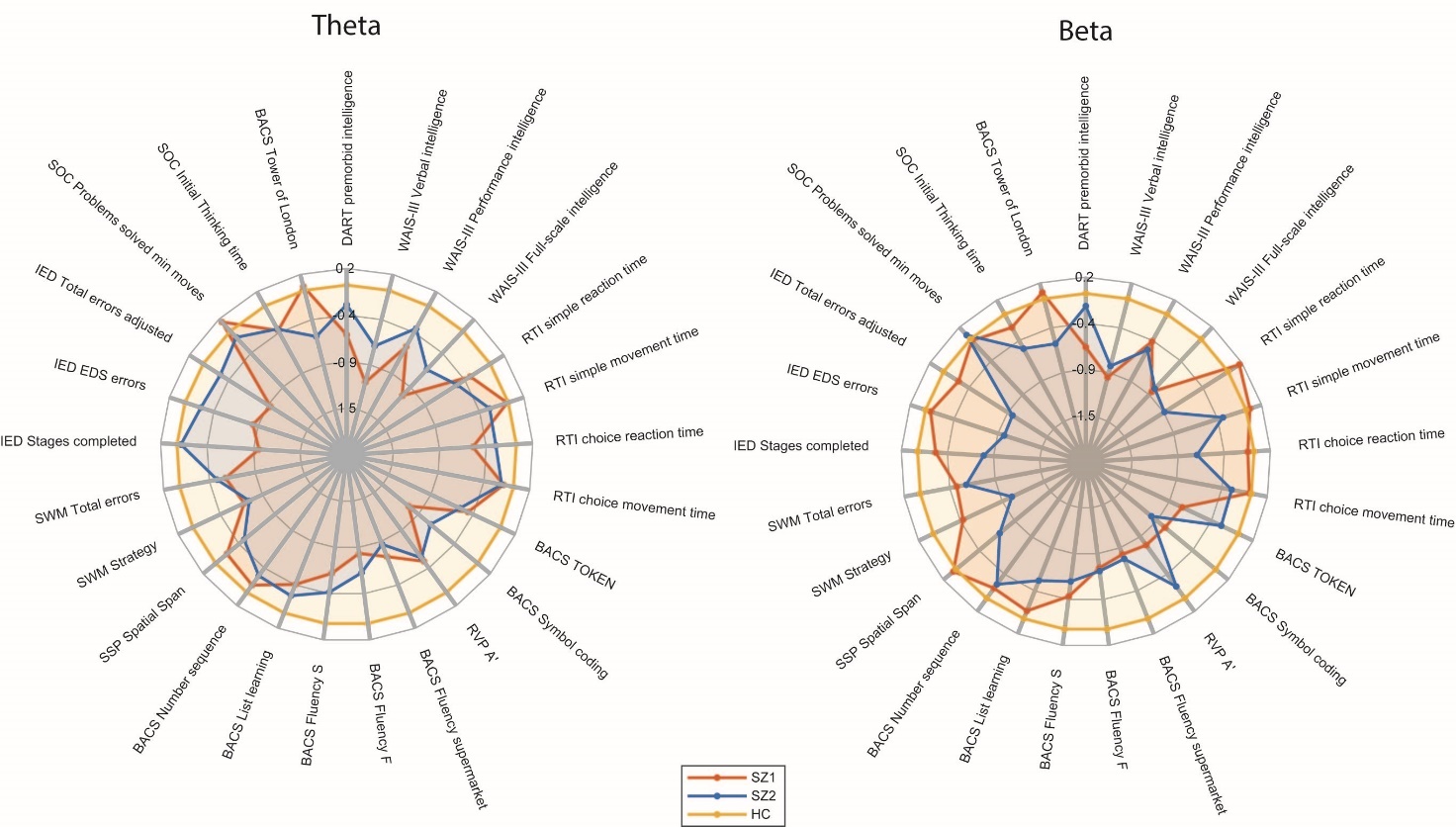


Supplementary Figure S7: Cognitive profiles of the schizophrenia subgroups, subgroup 1 (SZ1, red) and subgroup 2 (SZ2, blue), derived using the theta frequency band (left) and the beta frequency band (right). The scores are converted to z-scores based on the HC and some of the measures are inverted to reflect that higher scores are better. No differences between the subgroups survived correction for multiple comparison. See Table 2 and Supplementary Tables S4-S7 for details.

# Supplementary Tables

Supplementary Table S1: Comparison of the theta subgroups on PANSS items. Significant differences are in bold. No differences survived correction for multiple comparison.


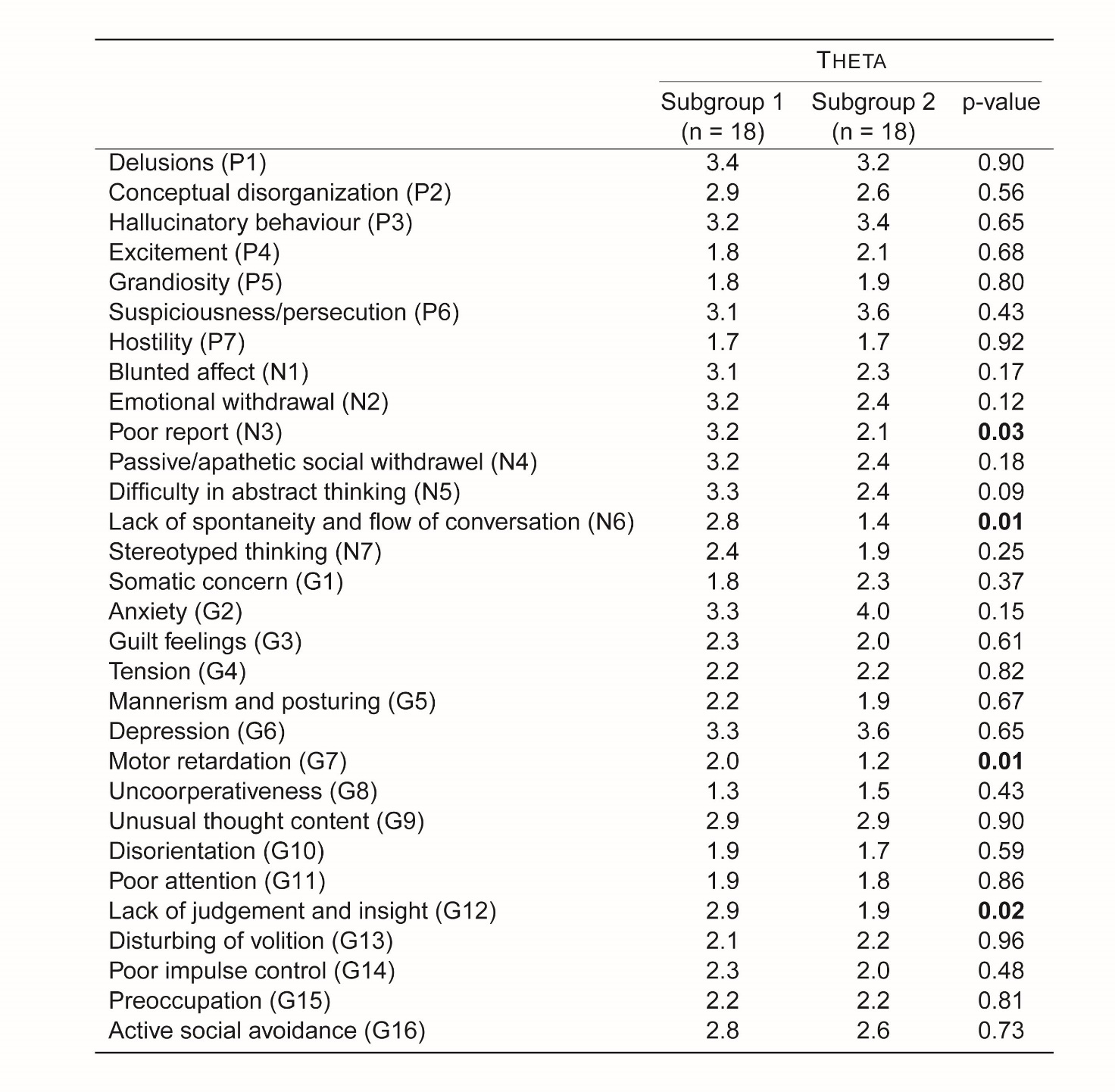


Supplementary Table S2: Comparison of the beta subgroups on PANSS items. Significant differences are in bold. No differences survived correction for multiple comparison.


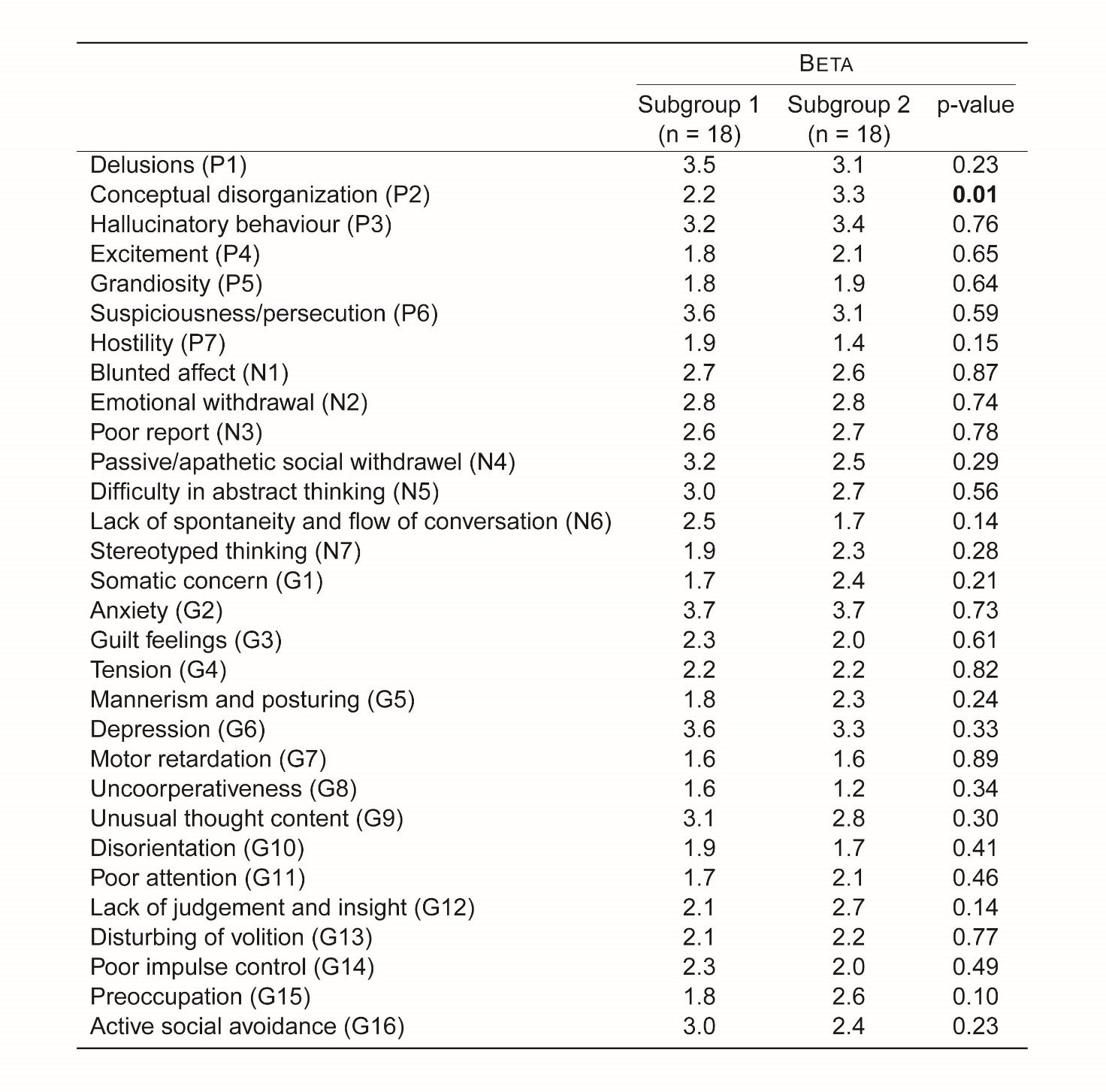


Supplementary Table S3: Comparison of the patients with schizophrenia (SZ) and the healthy controls (HC) on functional connectivity. Significant differences are in bold. No differences survived correction for multiple comparison within each frequency and using the false discovery rate (FDR).
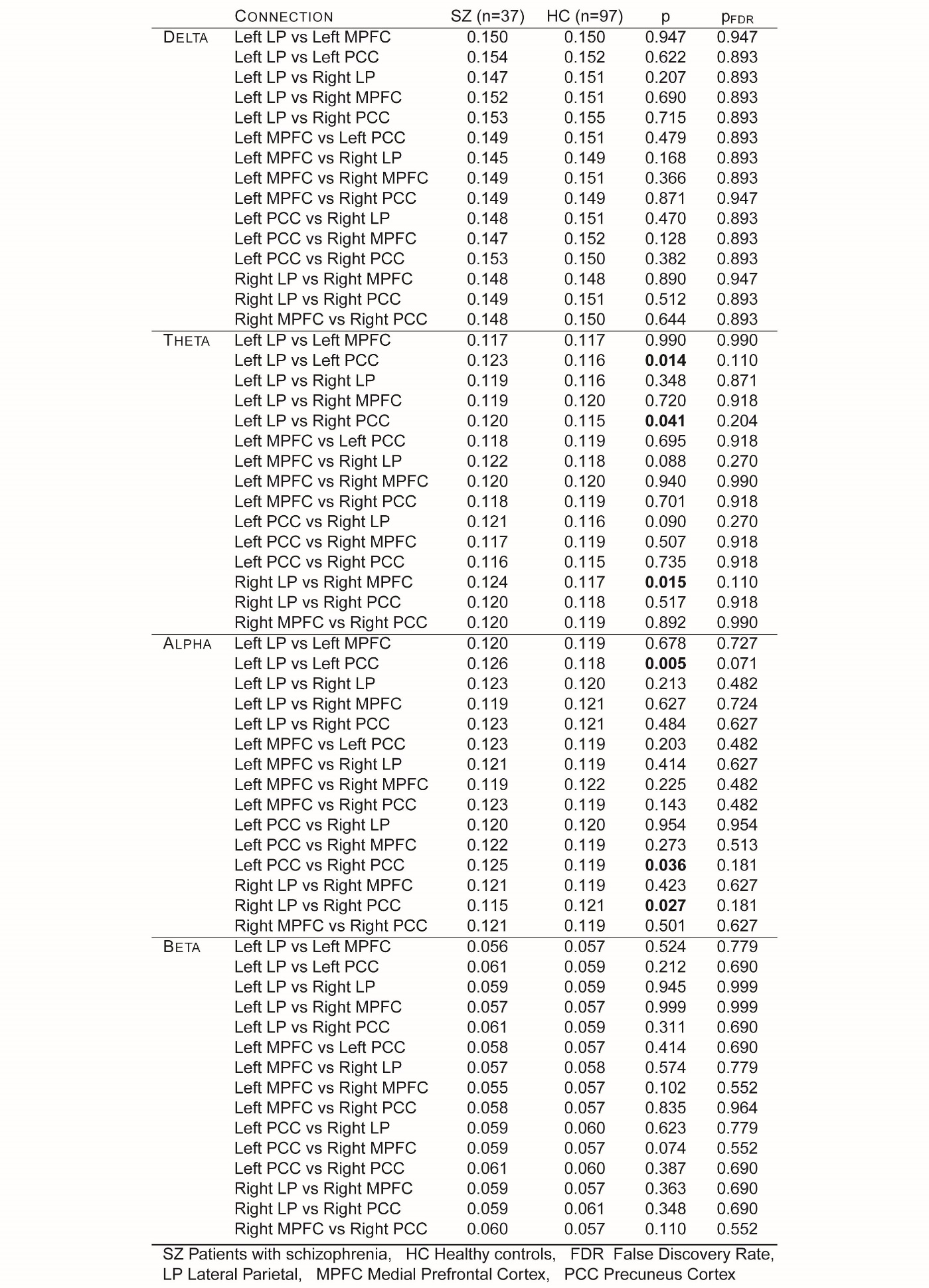


*Supplementary Table S4: Comparison of Theta Subgroup 1 (SZ 1) and healthy controls (HC) on cognition and functional connectivity.
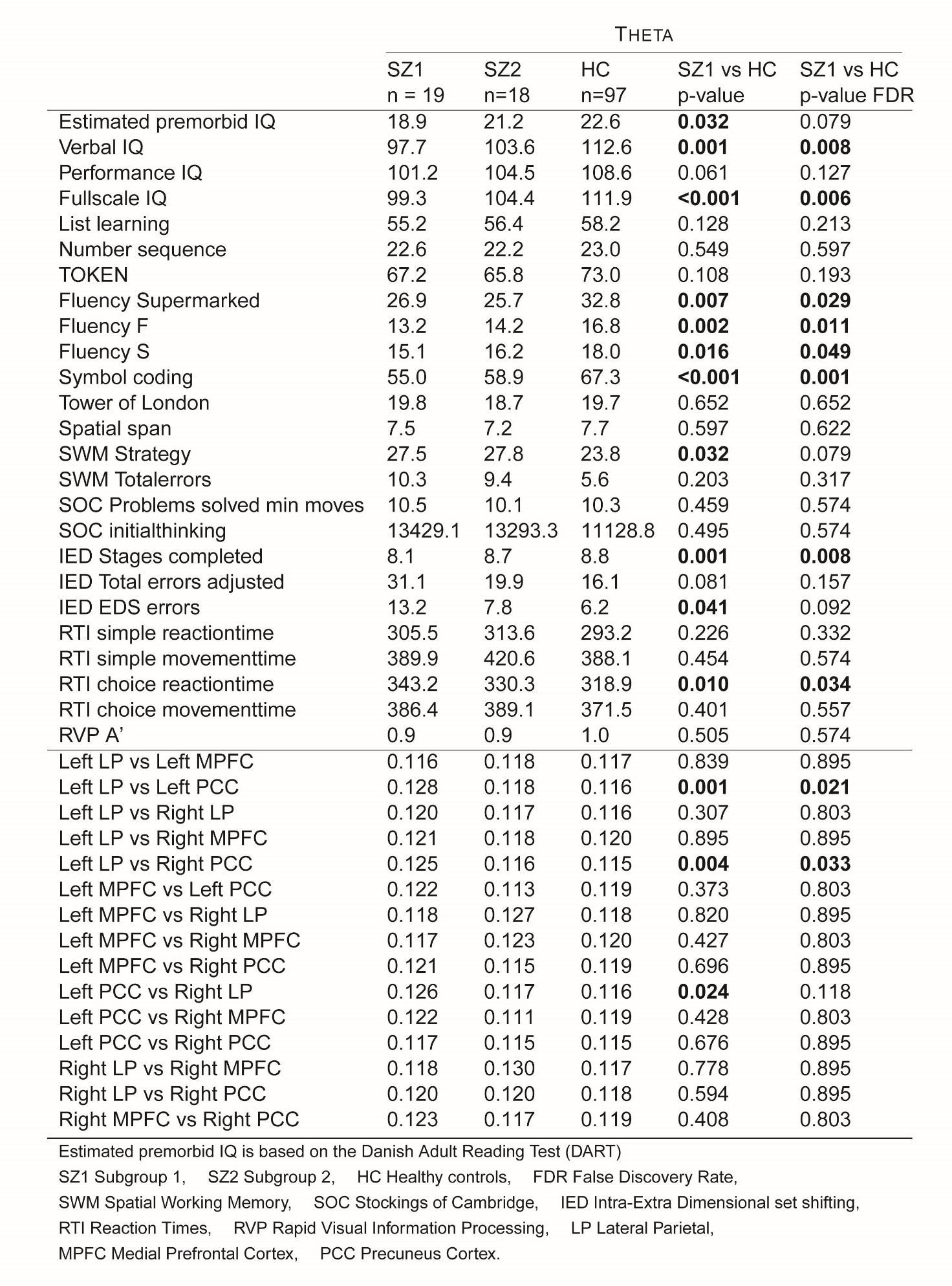
*

*Supplementary Table S5: Comparison of Theta Subgroup 2 (SZ 2) and healthy controls (HC) on cognition and functional connectivity.
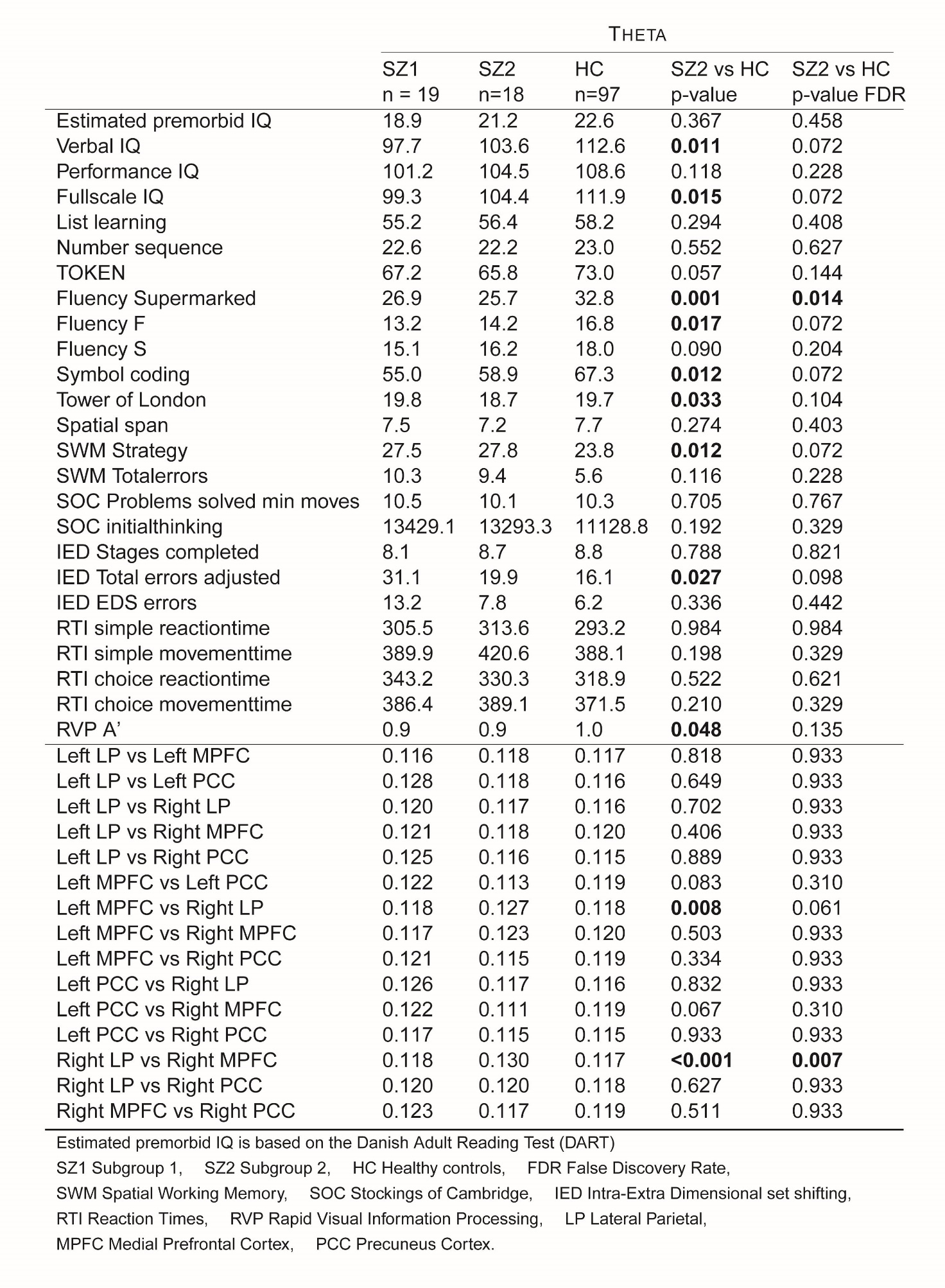
*

*Supplementary Table S6: Comparison of Beta Subgroup 1 (SZ 1) and healthy controls (HC) on cognition and functional connectivity.
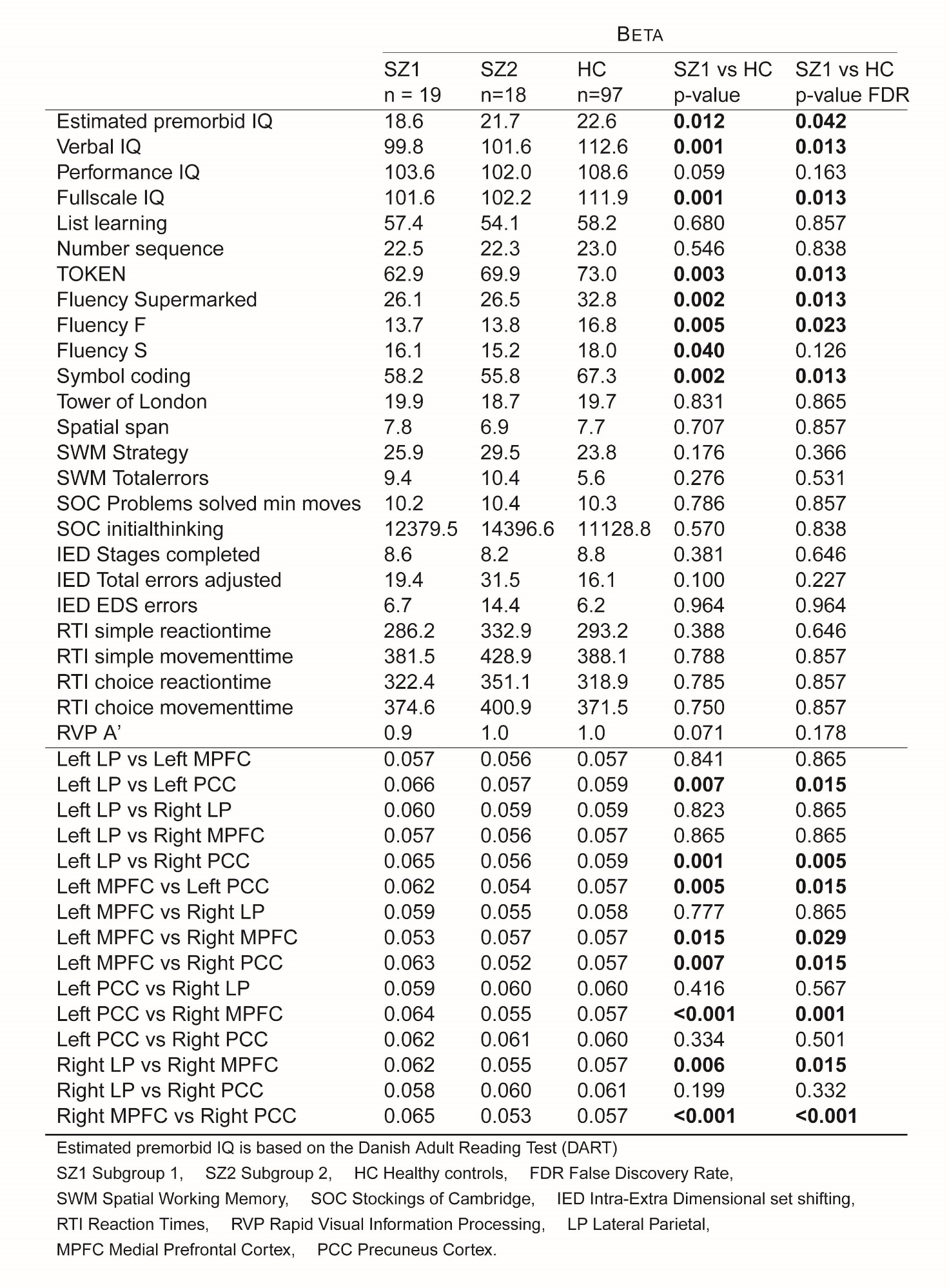
*

*Supplementary Table S7: Comparison of Beta Subgroup 2 (SZ 2) and healthy controls (HC) on cognition and functional connectivity.*

*
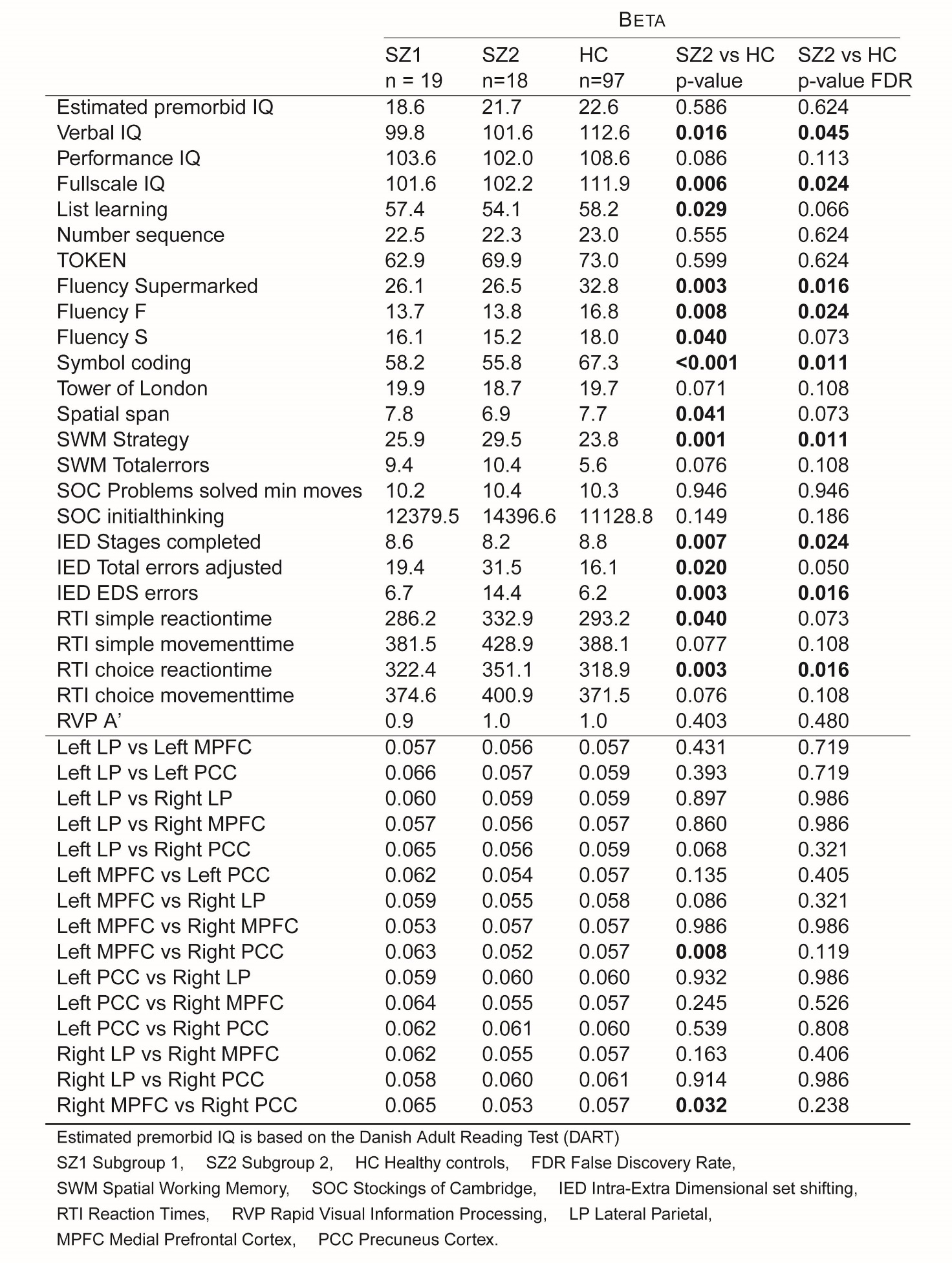
*
